# Cross-classification between self-rated health and health status: longitudinal analyses of all-cause mortality and leading causes of death in the UK

**DOI:** 10.1038/s41598-021-04016-x

**Published:** 2022-01-10

**Authors:** Julian Mutz, Cathryn M. Lewis

**Affiliations:** 1 Social, Genetic and Developmental Psychiatry Centre, Institute of Psychiatry, Psychology & Neuroscience, King’s College London, Memory Lane, London, SE5 8AF UK; 2 Department of Medical and Molecular Genetics, Faculty of Life Sciences & Medicine, King’s College London, London, UK

**Keywords:** Diseases, Medical research, Risk factors

## Abstract

Risk stratification is an important public health priority that is central to clinical decision making and resource allocation. The aim of this study was to examine how different combinations of self-rated and objective health status predict all-cause mortality and leading causes of death in the UK. The UK Biobank study recruited > 500,000 participants between 2006 and 2010. Self-rated health was assessed using a single-item question and health status was derived from medical history, including data on 81 cancer and 443 non-cancer illnesses. Analyses included > 370,000 middle-aged and older adults with a median follow-up of 11.75 (IQR = 1.4) years, yielding 4,320,270 person-years of follow-up. Compared to individuals with excellent self-rated health and favourable health status, individuals with other combinations of self-rated and objective health status had a greater mortality risk, with hazard ratios ranging from HR = 1.22 (95% CI 1.15–1.29, *P*_Bonf._ < 0.001) for individuals with good self-rated health and favourable health status to HR = 7.14 (95% CI 6.70–7.60, *P*_Bonf._ < 0.001) for individuals with poor self-rated health and unfavourable health status. Our findings highlight that self-rated health captures additional health-related information and should be more widely assessed. The cross-classification between self-rated health and health status represents a straightforward metric for risk stratification, with applications to population health, clinical decision making and resource allocation.

## Introduction

Self-rated health is used extensively in epidemiological and public health research, and many studies have shown that it predicts morbidity and mortality^1^. It is a single item measure of subjective health status and likely encompasses biological, psychological, functional and socio-economic dimensions, including quality of life. The relation between self-rated health and mortality may, however, differ between populations. For example, poor self-rated health is more predictive of mortality in men than in women^2,3^, self-rated health is more predictive of mortality in individuals of low socio-economic status^4^ and there is evidence that the association between self-rated health and mortality differs between regions in the UK^5,6^.

Despite its simplicity, self-rated health is correlated with objective assessments of health^7^. For example, research in > 16,000 Chinese residents aged 18–80 found a higher prevalence of diseases and abnormalities in laboratory tests in individuals with poor self-rated health^8^. Similarly, longitudinal data from the French Gazel cohort suggested that general health status was associated with a wide range of diseases, and that changes in participants’ self-rated health were associated with subsequent differences in reported diseases^9^. A study of 1,322 community-dwelling elderly aged 60 or older who participated in the Bambuí Cohort Study of Aging in Brazil examined how well self-rated health predicted 10-year mortality, compared to a comprehensive health score derived from objective clinical measures^10^. Individuals with poor self-rated health had a two-fold increased risk of death during the follow-up period, compared to individuals who rated their health as good or excellent. Self-rated health was comparable to the health score in predicting mortality and remained predictive of mortality after adjustment for the health score, suggesting that it captures additional health-related information.

Electronic health records and other patient data that are available to health care providers could be used to derive a measure of overall health status. Self-rated health can be assessed using a single-item question and is for instance included in census questionnaires^11^ but not, to our knowledge, routinely collected during primary care registrations or visits. The cross-classification between self-rated health and objective health status based on medical history or clinical measures could represent a readily available metric for risk stratification. The identification of at-risk populations is an important public health priority that is central to clinical decision making and resource allocation.

The aim of the present study was to examine self-rated health and health status based on medical history to predict mortality in middle-aged and older adults in the UK Biobank. We created a matrix of the cross-classification between self-rated health and health status and examined how different combinations of self-rated and objective health status predicted (i) all-cause mortality and (ii) cause-specific mortality from leading causes of death in the UK^12^ during a follow-up period of approximately 12 years.

We hypothesised that individuals with concordant self-rated health and health status would demonstrate the longest (favourable health status and good or excellent self-rated health) and shortest (unfavourable health status and poor or fair self-rated health) survival times, respectively. Individuals with discordant self-rated health and health status were predicted to demonstrate intermediate survival times, although no predictions were made about the precise order of survival times (Supplement 1). The aim of comparing these survival times was to explore whether the discordant categories would provide additional information about the relative importance of self-rated health and health status with respect to future health outcomes.

This study contributes to the evidence base for self-rated health as a measure for risk stratification and prediction of mortality by (i) using a large sample of middle-aged and older adults with a long period of follow-up, thereby reducing the risk of random error, (ii) controlling for multiple socio-demographic and lifestyle factors that could potentially confound the association between self-rated health and mortality, (iii) examining leading causes of death in the UK, in addition to all-cause mortality, and (iv) for the first time examining the combination of self-rated health and a life insurance measure of overall health status in relation to mortality.

## Methods

### Study population

The UK Biobank is a prospective study of > 500,000 UK residents aged 37–73 at baseline, recruited between 2006 and 2010. Details of the study rationale and design have been reported elsewhere^13^. Briefly, individuals registered with the UK National Health Service (NHS) and living within a 25-mile (~ 40 km) radius of one of 22 assessment centres were invited to participate (9,238,453 postal invitations sent). At the baseline assessment, participants completed electronic questionnaires and nurse-led interviews to provide data on sociodemographic characteristics, health behaviours and their medical history. Linked hospital inpatient records are available for most participants and these data have been linked to death registries. Due to its scale and breadth of data collection, the UK Biobank represents an unprecedented resource to investigate the determinants of health and disease.

### Exposures

Data on 81 cancer and 443 non-cancer illnesses (past and current) were ascertained through touchscreen self-report questionnaire and confirmed during a verbal interview by a trained nurse. In order to provide a single health indicator (“health status”) based on a previously defined algorithm, we used a classification developed by the Reinsurance Group of America (RGA). An experienced underwriter classified each illness according to whether it was “likely acceptable for standard life insurance” based on its associated mortality risk^14^. Participants were thus classified as having favourable or unfavourable health status based on their reported cancer and non-cancer illnesses. Details of this classification have been reported previously^14,15^.

Participants’ self-rated health was assessed using the question “In general how would you rate your overall health?”. Response options included “Poor”, “Fair”, “Good” and “Excellent”.

We derived a measure of the cross-classification between health status and self-rated health. Individuals with missing data or who responded “prefer not to answer” or “do not know” to the self-rated health question were excluded.

### Ascertainment of mortality

Our primary outcome was all-cause mortality, i.e., death from any cause. All-cause mortality represents a standard index used in clinical decision making that is easily assessed and concrete^16^. The date of death was obtained through linkage with national death registries from NHS Digital for participants in England and Wales and from the NHS Central Register for participants in Scotland. The censoring date for mortality was 30 November 2020. The most recent death was recorded for 18 December 2020, although data were not complete for December 2020.

We also examined cause-specific mortality for leading causes of death in the UK^12^. The primary cause of death was recorded based on the International Classification of Diseases (10th revision). The following outcomes were examined: ischaemic heart diseases (I20-I25), cerebrovascular diseases (I60-I69), influenza and pneumonia (J09-J18), dementia and Alzheimer’s disease (F01, F03 and G30), chronic lower respiratory diseases (J40-J47) and malignant neoplasms (C00-C97). For each cause-specific death, individuals who died of other causes were censored at their age at death.

### Covariates

Potential confounders of the association between self-rated health or health status and all-cause or cause-specific mortality were identified from the baseline assessment data: sociodemographic factors (sex, ethnicity [6 levels], highest educational/professional qualification [4 levels]^17^ and annual household income [5 levels]) and lifestyle (smoking [3 levels], alcohol intake frequency [6 levels], physical activity [number of days per week spent walking, engaging in moderate-intensity physical activity or engaging in vigorous-intensity physical activity for ≥ 10 min continuously]). All covariates were assumed to be fixed.

### Exclusion criteria

Participants for whom their genetic sex, inferred from the genotype information on the Y and X chromosomes, and self-reported sex did not match were excluded. Individuals with missing data or who responded “do not know” or “prefer not to answer” to any of the assessed covariates were also excluded from analyses.

### Statistical analyses

Analyses were pre-specified prior to inspection of the data (preregistration: osf.io/x38cq) and algorithms were tested on simulated data. Statistical analyses and data visualisation were done in R (version 3.6.0).

Characteristics of the full and analytical sample were summarised using means and standard deviations or counts and percentages. The total number of self-reported diseases and the frequencies of illnesses by disease group were summarised for each level of the cross-classification between health status and self-rated health.

We present the number of individuals who died during follow-up of any cause (all-cause mortality) and of specific causes (cause-specific mortality). The minimum number of observed deaths was set a priori at 20 for each level of our primary exposure. This criterion was based on a previous UK Biobank study of 5-year mortality^18^. Finally, we calculated person-years of follow-up and the median duration of follow-up of censored individuals.

Unadjusted survival probabilities by health status, self-rated health and health cross-classification were estimated non-parametrically from observed survival times using the Kaplan–Meier (KM) method^19^. We present KM survival curves and *p*-values from log-rank tests. Hazard ratios (HRs) and 95% confidence intervals were estimated using Cox proportional hazards models^20^ to examine associations between the health cross-classification and mortality adjusted for potential confounders. Age (in years) was used as the underlying time axis, conditional on living to age 40. We fitted a sequence of three models: Model 1 – unadjusted; Model 2 – adjusted for sociodemographic characteristics; Model 3 – additionally adjusted for lifestyle. We present plots of the estimated survival probability and cumulative hazards. Continuous covariates were fixed at their mean value while categorical covariates were fixed at the largest group. When presenting results in tables and figures, the levels of the health cross-classification are shown in ascending order by their HR for all-cause mortality from the results of Model 1. Scaled Schoenfeld residuals and Log(-log(survival)) curves as a function of time were examined to assess the assumption of proportional hazards. Martingale residuals were visually inspected to assess the assumption of log-linearity for continuous covariates.

Adjusted *P*-values were calculated using the p.adjust() command in R to account for multiple testing across models, separately for each outcome. *P*-values were corrected for 21 tests (three models × seven estimated parameters). Two methods were used: (1) Bonferroni and (2) Benjamini & Hochberg^21^, all two-tailed, with *α* = 0.05 and false discovery rate of 5%, respectively.

### Additional analyses

Given that there are differences in self-rated health, medical diagnoses and mortality between males and females, we repeated our main analysis of all-cause mortality stratified by sex. In a sensitivity analysis we examined a simplified health cross-classification in which fair and poor self-rated health and good and excellent self-rated health were merged prior to analysis (Supplement 1). Finally, we also conducted a sensitivity analysis in which we limited the follow up to the period prior to 30 January 2020 to assess any impact of the COVID-19 pandemic. The analyses stratified by sex and restricted to the period prior to 30 January 2020 were not pre-registered.

### Ethics approval

Ethical approval for the UK Biobank study has been granted by the National Information Governance Board for Health and Social Care and the NHS North West Multicentre Research Ethics Committee (11/NW/0382). No project-specific ethical approval is needed. Data access permission has been granted under UK Biobank application 45514. Participants provided informed consent to use of their de-identified data. The authors confirm that this study was carried out in accordance with relevant guidelines and regulations.

## Results

### Sample characteristics

Of the 502,521 UK Biobank participants, 487,195 (96.95%) had complete data on self-rated health and health status. We retained an analytical sample of 373,761 participants after removing individuals with missing data on covariates (*n* = 118 190), who did not meet our inclusion criteria (*n* = 372) or whose recorded date of death was before or on the same day as the baseline assessment (*n* = 495) (Supplement Fig. 1).

Descriptive statistics of the full and analytical samples are presented in Supplement Table 1. The mean age at the baseline assessment of participants included in our analytical sample was 56.02 (SD = 8.08) years and 51.8% of these participants were female. We classified 117,212 (31.36%) participants as having an unfavourable health status and 256,549 (68.64%) participants as having a favourable health status. Most participants (*n* = 219,628, 58.76%) had good self-rated health and 14,185 (3.80%), 73,138 (19.57%) and 66,810 (17.88%) participants had poor, fair and excellent self-rated health, respectively (Table 1).

**Table 1:** Cross-classification of health status and self-rated health.

health status	self-rated health			
	poor *n* = 14185	fair *n* = 73138	good *n* = 219628	excellent *n* = 66810
favourable *n* = 256549	4004 (1.07%)	37863 (10.13%)	159173 (42.59%)	55509 (14.85%)
unfavourable *n* = 117212	10181 (2.72%)	35275 (9.44%)	60455 (16.17%)	11301 (3.02%)

Percentages in cells are based on complete analytical sample of *n* = 373,761.

The average number of illnesses by health cross-classification ranged from 0.73 (SD = 0.99) in individuals with excellent self-rated health and favourable health status to 4.93 (SD = 2.70) in individuals with poor self-rated health and unfavourable health status (Supplement Table 2). The distributions of the number of illnesses by health cross-classification are presented in Supplement Fig. 2 and show that there were substantial differences between groups. The proportions of individuals with at least one non-cancer or cancer illness within several broader illness groups are presented by health cross classification in Supplement Fig. 3 and Supplement Fig. 4, respectively.

The median follow-up was 11.75 (IQR = 1.4) years, yielding 4,320,270 person years of follow-up. The median follow-up of censored individuals was 11.81 (IQR = 1.35) years and the potential median survival time, calculated using the reverse Kaplan–Meier method, was 11.819 (95% CI = 11.81–11.82) years. We observed 21,980 (5.88%) deaths from any cause. Deaths observed for specific causes were 2,432 (0.65%) for ischaemic heart disease, 908 (0.24%) for cerebrovascular disease, 409 (0.11%) for influenza and pneumonia, 701 (0.19%) for dementia and Alzheimer’s disease, 699 (0.19%) for chronic lower respiratory disease and 11,171 (2.99%) for malignant neoplasms.

### All-cause mortality

**Figure 1:**
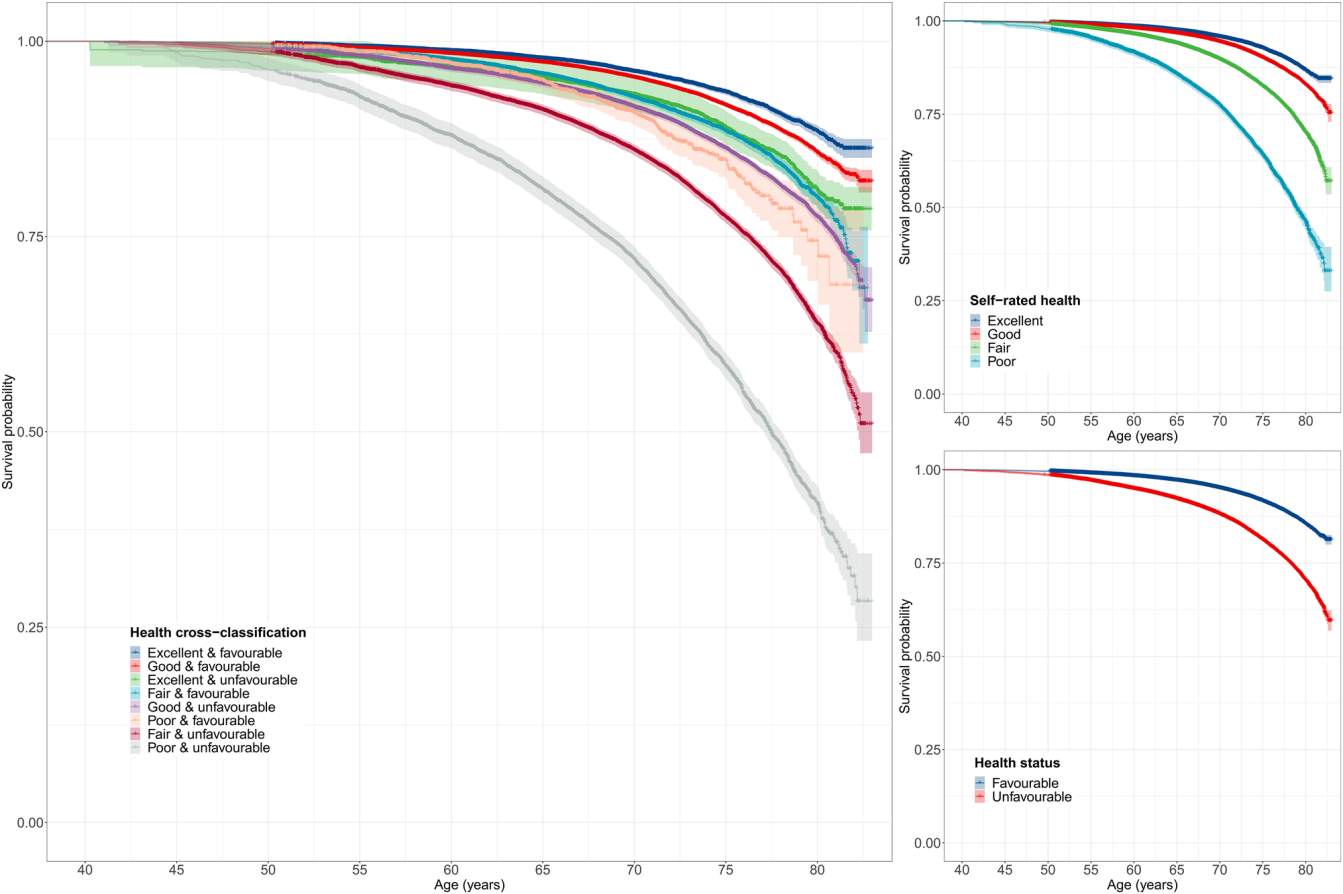
Kaplan–Meier survival probabilities for all-cause mortality. Full health cross-classification, self-rated health and health status. 89 observations were above graph maximum of age 83.

Kaplan–Meier survival probabilities for all-cause mortality are presented in Fig. 1. Favourable health status and better self-rated health were associated with longer survival times (log-rank test *P*-values < 0.001). Different levels of the cross-classification between health status and self-rated health were associated with varying survival times (log-rank test *P* < 0.001). We found no evidence that survival probabilities differed by year of attending the baseline assessment *P* = 0.15).

Unfavourable health status was associated with greater hazards of all-cause mortality than favourable health status, with hazard ratios ranging from HR = 2.05 (95% CI 1.99–2.10, *P*_Bonf__._< 0.001) in Model 1 to HR = 1.85 (95% CI 1.80–1.90, *P*_Bonf._ < 0.001) in Model 3 (Supplement Table 3).

Poor, fair and good self-rated health were associated with higher hazards of all-cause mortality than excellent self-rated health across Models 1–3. The largest estimate was HR = 5.79 (95% CI 5.47–6.12, *P*_Bonf._ < 0.001) for poor compared to excellent selfrated health in Model 1 (Supplement Table 4).

Results of the Cox proportional hazards Models 1–3 for the health cross-classification are presented in Table 2. Compared to excellent self-rated health and favourable health status, all other levels of the full health cross-classification were associated with greater hazards, ranging from HR = 1.22 (95% CI 1.15–1.29, *P*_Bonf._) for good self-rated health and favourable health status to HR = 7.14 (95% CI 6.70–7.60, P_Bonf__._ < 0.001) for poor self-rated health and unfavourable health status. The order of these levels was consistent across the three models, suggesting that individuals with concordant favourable self-rated health and health status had the lowest hazard and those with concordant unfavourable self-rated health and health status had the highest hazard. Participants with discordant self-rated health and health status had intermediate hazards. Compared to the results from Model 1, we observed attenuation in the hazard ratios from Models 2 and 3, although the overall pattern of results was consistent across these analyses (all Bonferroni-adjusted *P*-values < 0.001). Survival probabilities and cumulative hazards for Model 3 are presented in Supplement Fig. 5.

**Table 2:** Cox proportional hazards model: all-cause mortality.

Health cross-classification		Model 1			Model 2			Model 3		
		HR	95% CI		HR	95% CI		HR	95% CI	
Excellent	Favourable	Ref	–	–	Ref	–	–	Ref	–	–
Good	Favourable	1.22	1.15	1.29	1.20	1.14	1.27	1.16	1.10	1.23
Excellent	Unfavourable	1.42	1.30	1.55	1.38	1.26	1.51	1.37	1.25	1.50
Fair	Favourable	1.86	1.74	1.99	1.77	1.65	1.89	1.59	1.49	1.70
Good	Unfavourable	1.87	1.77	1.98	1.81	1.71	1.91	1.73	1.63	1.83
Poor	Favourable	2.79	2.42	3.22	2.62	2.27	3.02	2.17	1.88	2.51
Fair	Unfavourable	3.26	3.08	3.45	3.02	2.85	3.19	2.72	2.57	2.88
Poor	Unfavourable	7.14	6.70	7.60	6.34	5.94	6.76	5.24	4.90	5.61

HR = hazard ratio; CI = confidence interval. Model 1—unadjusted; Model 2—adjusted for sociodemographic characteristics; Model 3 – additionally adjusted for lifestyle factors. All Bonferroni-adjusted *P*-values < 0.001.

### Cause-specific mortality

Survival probabilities for specific causes of death are presented in Supplement Fig. 6. Results from Cox proportional hazards Models 1–3 are presented in Table 3 and Supplement Table 5. Compared to participants with excellent self-rated health and favourable health status, all other levels of the health cross-classification were associated with higher hazards. For example, HRs for ischaemic heart diseases from Model 3 ranged from HR = 1.14 (95% CI 0.96–1.35, *P*_BH_ = 0.12) for good self-rated health and favourable health status to HR = 5.11 (95% CI 4.17–6.26, *P*_Bonf._ < 0.001) for poor self-rated health and unfavourable health status. We generally observed attenuation in the hazard ratios from Models 2 and 3 compared to those from Model 1. For example, the highest hazard ratio for malignant neoplasm was HR = 7.02 (95% CI 6.43–7.67, P_Bonf._ < 0.001) for poor self-rated health and unfavourable health status in Model 1, HR = 6.24 (95% CI 5.70–6.83, *P*_Bonf._ < 0.001) in Model 2 and HR = 5.20 (95% CI 4.73–5.71, *P*_Bonf._ < 0.001) in Model 3. The overall pattern of results was similar to the results for all-cause mortality, although there was less evidence of differences for causes of death with lower numbers of observed deaths (cerebrovascular diseases, influenza and pneumonia and dementia and Alzheimer’s disease) and levels of the health cross-classification that were associated with longer survival times than good self-rated health and unfavourable health status.

**Table 3:** Cox proportional hazards model: cause-specific mortality.

Health cross-classification		Model 1			Model 2			Model 3		
		HR	95% CI		HR	95% CI		HR	95% CI	
**Ischaemic heart diseases**										
Excellent	Favourable	Ref	–	–	Ref	–	–	Ref	–	–
Good	Favourable	1.21	1.02	1.43	1.19	1.01	1.41	1.14	0.97	1.35
Excellent	Unfavourable	1.51	1.16	1.98	1.47	1.12	1.92	1.45	1.11	1.90
Fair	Favourable	2.16	1.77	2.63	2.03	1.67	2.48	1.81	1.48	2.21
Good	Unfavourable	1.84	1.55	2.18	1.77	1.49	2.10	1.68	1.42	2.00
Poor	Favourable	3.71	2.53	5.43	3.44	2.35	5.05	2.81	1.91	4.14
Fair	Unfavourable	3.38	2.85	4.01	3.10	2.60	3.68	2.76	2.32	3.29
Poor	Unfavourable	7.14	5.90	8.66	6.28	5.16	7.64	5.11	4.17	6.26
**Cerebrovascular diseases**										
Excellent	Favourable	Ref	–	–	Ref	–	–	Ref	–	–
Good	Favourable	1.25	0.93	1.66	1.25	0.94	1.67	1.22	0.91	1.62
Excellent	Unfavourable	1.67	1.06	2.61	1.63	1.04	2.55	1.61	1.03	2.53
Fair	Favourable	2.13	1.52	3.00	2.09	1.48	2.95	1.90	1.34	2.69
Good	Unfavourable	2.20	1.65	2.94	2.16	1.62	2.89	2.09	1.56	2.79
Poor	Favourable	3.24	1.61	6.53	3.16	1.56	6.39	2.64	1.30	5.36
Fair	Unfavourable	3.91	2.92	5.22	3.73	2.78	5.00	3.41	2.53	4.59
Poor	Unfavourable	9.49	6.93	13.01	8.75	6.34	12.07	7.19	5.15	10.03
**Influenza/pneumonia**										
Excellent	Favourable	Ref	–	–	Ref	–	–	Ref	–	–
Good	Favourable	1.22	0.82	1.82	1.17	0.79	1.75	1.11	0.75	1.66
Excellent	Unfavourable	1.13	0.55	2.3	1.09	0.53	2.22	1.07	0.53	2.19
Fair	Favourable	1.32	0.77	2.25	1.19	0.70	2.04	1.05	0.61	1.80
Good	Unfavourable	2.17	1.46	3.24	2.02	1.35	3.01	1.90	1.27	2.84
Poor	Favourable	4.13	1.72	9.91	3.63	1.51	8.74	2.96	1.22	7.18
Fair	Unfavourable	2.74	1.81	4.17	2.40	1.57	3.66	2.11	1.38	3.25
Poor	Unfavourable	6.92	4.36	10.98	5.71	3.56	9.16	4.66	2.86	7.58
**Dementia/Alzheimer's disease**										
Excellent	Favourable	Ref	–	–	Ref	–	–	Ref	–	–
Good	Favourable	1.25	0.92	1.70	1.24	0.91	1.68	1.19	0.87	1.62
Excellent	Unfavourable	1.28	0.76	2.17	1.24	0.73	2.10	1.23	0.72	2.07
Fair	Favourable	1.44	0.96	2.15	1.38	0.92	2.07	1.23	0.82	1.85
Good	Unfavourable	2.02	1.48	2.75	1.95	1.43	2.66	1.85	1.35	2.53
Poor	Favourable	2.87	1.30	6.32	2.79	1.26	6.16	2.29	1.03	5.09
Fair	Unfavourable	3.22	2.34	4.41	3.00	2.18	4.13	2.67	1.93	3.70
Poor	Unfavourable	7.00	4.91	9.99	6.35	4.42	9.14	5.16	3.54	7.51
**Chronic lower respiratory disease**										
Excellent	Favourable	Ref	–	–	Ref	–	–	Ref	–	–
Good	Favourable	1.50	1.07	2.09	1.46	1.04	2.04	1.42	1.01	1.98
Excellent	Unfavourable	2.52	1.58	4.03	2.46	1.54	3.94	2.41	1.51	3.86
Fair	Favourable	2.38	1.6	3.54	2.19	1.47	3.26	1.99	1.33	2.97
Good	Unfavourable	2.35	1.67	3.30	2.24	1.59	3.16	2.14	1.52	3.01
Poor	Favourable	4.01	1.88	8.55	3.58	1.67	7.65	2.99	1.39	6.41
Fair	Unfavourable	4.09	2.90	5.76	3.68	2.60	5.21	3.30	2.33	4.69
Poor	Unfavourable	8.10	5.51	11.9	6.96	4.71	10.31	5.80	3.87	8.68
**Malignant neoplasm**										
Excellent	Favourable	Ref	–	–	Ref	–	–	Ref	–	–
Good	Favourable	1.19	1.10	1.29	1.18	1.09	1.27	1.14	1.06	1.23
Excellent	Unfavourable	1.31	1.15	1.49	1.27	1.12	1.45	1.26	1.11	1.44
Fair	Favourable	1.81	1.65	1.99	1.72	1.57	1.89	1.55	1.41	1.71
Good	Unfavourable	1.84	1.70	1.99	1.78	1.65	1.93	1.71	1.58	1.85
Poor	Favourable	2.40	1.94	2.96	2.25	1.82	2.79	1.88	1.52	2.33
Fair	Unfavourable	3.20	2.96	3.46	2.96	2.74	3.21	2.68	2.48	2.91
Poor	Unfavourable	7.02	6.43	7.67	6.24	5.70	6.83	5.20	4.73	5.71

HR = hazard ratio; CI = confidence interval; Bonferroni and Benjamini & Hochberg adjusted *P*-values are presented in Supplement Table 5. Model 1—unadjusted; Model 2—adjusted for sociodemographic characteristics; Model 3—additionally adjusted for lifestyle factors.

### Additional analyses

**Figure 2:**
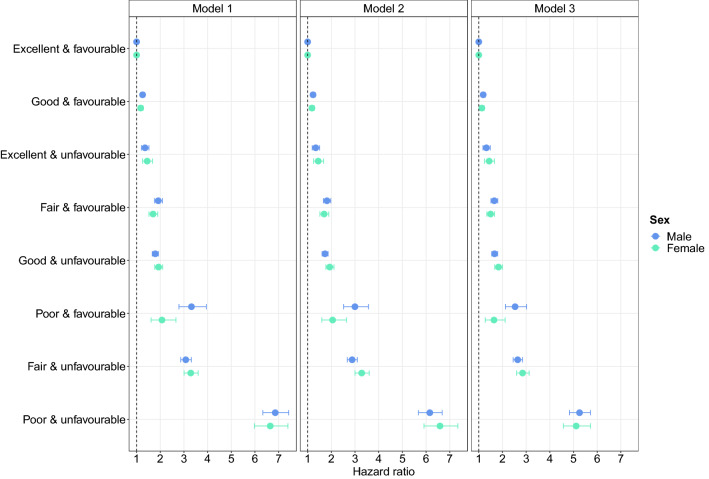
All-cause mortality by health cross-classification stratified by sex. Hazard ratios (HR) with 95% confidence intervals from Cox proportional hazards models. Model 1—unadjusted; Model 2—adjusted for sociodemographic characteristics (excluding sex); Model 3—additionally adjusted for lifestyle factors.

Analyses stratified by sex are presented in Fig. 2 and Supplement Table 6. Although the hazard ratios were fairly similar in both sexes, there was some evidence that females with unfavourable health status had slightly higher hazards for any level of self-rated health, relative to females with excellent self-rated health and favourable health status, than males with unfavourable health status and any level of self-rated health, relative to males with excellent self-rated health and favourable health status. The reverse pattern was observed for favourable health status and self-rated health.

For the simplified health cross-classification, the average number of illnesses was 1.07 (SD = 1.22) in individuals with good/excellent self-rated health and favourable health status, 2.78 (SD = 1.67) in individuals with good/excellent self-rated health and unfavourable health status, 1.86 (SD = 1.59) in individuals with poor/fair self-rated health and favourable health status and 4.00 (SD = 2.30) in individuals with poor/fair self-rated health and unfavourable health status.

Kaplan–Meier survival probabilities from this sensitivity analysis are presented in Supplement Fig. 7, suggesting that different levels of the simplified health cross-classification were associated with varying survival times (log-rank test *P<* 0.001). We found that participants with favourable health status and good or excellent self-rated health had the longest survival times, while participants with unfavourable health status and poor or fair self-rated health had the shortest survival times. Participants with discordant health status and self-rated health had intermediate survival times. Individuals with good or excellent self-rated health but unfavourable health status, i.e., for whom their objective assessment of health was worse, had only slightly shorter survival times than participants with favourable health status but fair or poor self-rated health.

Results from Cox proportional hazards Models 1–3 are presented in Table 4. Compared to favourable health status and good or excellent self-rated health, all other levels of the simplified health cross-classification were associated with increased hazards. Across Models 1 and 2, individuals with better self-rated health than health status had lower hazards than individuals with worse self-rated health than health status, compared to the reference group, although in Model 3 those with worse self-rated health than health status had lower hazards, similar to the Kaplan–Meier estimates. Individuals with unfavourable health status and self-rated health had the highest hazards across all models.

**Table 4:** Cox proportional hazards model: all-cause mortality.

Term		Model 1			Model 2			Model 3		
		HR	95% CI		HR	95% CI		HR	95% CI	
Good/excellent	Favourable	Ref	–	–	Ref	–	–	Ref	–	–
Good/excellent	Unfavourable	1.55	1.50	1.61	1.51	1.46	1.56	1.49	1.44	1.54
Poor/fair	Favourable	1.66	1.58	1.75	1.58	1.50	1.66	1.43	1.36	1.50
Poor/fair	Unfavourable	3.43	3.32	3.54	3.14	3.04	3.25	2.81	2.72	2.91

HR = hazard ratio; CI = confidence interval. Model 1—unadjusted; Model 2—adjusted for sociodemographic characteristics; Model 3—additionally adjusted for lifestyle factors. All Bonferroni-adjusted *P*-values < 0.001.

Limiting the follow up to the period prior to 30 January 2020 resulted in *n* = 2955 fewer participants and a sample size of *n* = 370,806 participants. There was little evidence of meaningful differences in the results for all-cause (Supplement Table 7) and cause-specific mortality (Supplement Table 8) for the full health cross-classification.

## Discussion

Using data from more than 370,000 UK Biobank participants with a median follow-up period of almost 12 years, we found that individuals with favourable health status or good to excellent self-rated health had a substantially lower risk of mortality than individuals with unfavourable health status or poor to fair self-rated health, respectively. The cross-classification between health status and self-rated health provided additional granularity to differentiate mortality risk, confirming that self-rated health captures health-related information beyond medical diagnoses and highlighting the potential value of combining these measures for risk stratification.

### Principal findings

As hypothesised, individuals with favourable health status and good to excellent self-rated health had the longest survival times, while individuals with unfavourable health status and poor to fair self-rated health had the shortest survival times. Individuals with discordant health status and self-rated health had intermediate survival times. For example, individuals with favourable health status based on their medical history but poor self-rated health had substantially shorter survival times than individuals with favourable health status and good to excellent self-rated health.

We observed similar results for leading causes of death in the UK^12^. However, there were some inconsistencies for causes of death with fewer observed deaths during the follow-up period. For influenza/pneumonia and dementia/Alzheimer’s disease, we did not find evidence of differences between the four health cross-classification levels with the longest survival times: (i) good self-rated health and favourable health status, (ii) excellent self-rated health and unfavourable health status and (iii) fair self-rated health and favourable health status, compared to (iv) excellent self-rated health and favourable health status.

In the sensitivity analysis in which we merged good and excellent self-rated health and poor and fair self-rated health prior to analysis, we observed that individuals with discordant health status and self-rated health had intermediate survival times, showing that both measures make important contributions to predicting all-cause mortality. Restricting the follow up to the period prior to 30 January 2020 did not suggest any meaningful impact of the COVID-19 pandemic on our results.

### Findings in context

A previous analysis of 5-year mortality in the UK Biobank identified self-rated health as the strongest predictor of all-cause mortality in men, out of 655 variables, and showed that self-rated health was consistently associated with cause-specific mortality^18^. Our analyses stratified by sex also provide indirect evidence that differences in self-rated health may have a greater impact on mortality in males than in females, consistent with previous research^2,3^. Our findings also support most previous research that found a graded association between self-rated health levels and mortality^1^. Findings from a Brazilian cohort study suggested that the 10-year mortality risk was similar for participants who reported fair or good to excellent self-rated health, compared to poor self-rated health^10^. However, the sample size was limited and the difference in results might be due to linguistic factors; ‘fair’ self-rated health might reflect average or normal health in Spanish or Portuguese^23^, while it might reflect less favourable health in English.

Although several previous studies have examined mortality outcomes associated with objective and subjective assessments of health^8,10,24^, no studies have, to our knowledge, examined the full cross-classification between health status and self-rated health for potential risk stratification. A recent study of 1259 older adults from Finland examined the combination of subjective and objective health in relation to all-cause mortality^25^, similar to our sensitivity analysis of the four-level health cross-classification. The authors found that participants who were subjectively and objectively healthy had the lowest mortality, while participants who were subjectively and objectively unhealthy had the highest mortality. However, there was little evidence that the discordant groups had a different mortality than the subjectively and objectively healthy group, contrary to our finding that these participants had intermediate survival times.

An analysis of nationally-representative data from >14,500 US adults found that individuals who reported better or worse self-rated health compared to their physicians assessment of general health (the agreement was 53.8%) had lower and higher mortality, respectively, over a median follow-up period of 13 years^26^. A potential limitation of this study was that physicians were not blinded to the participants’ self-rated health, although self-rated and physician-rated health were not highly correlated. Data from 710 Dutch men aged 64–84 years suggested that self-rated health and physician-rated health after a medical history assessment and physical examination independently predicted all-cause mortality over a follow-up period of 15 years^27^. However, this analysis suggested that in cases of discordant self-rated and physician-rated health, individuals whose physician evaluated their health less favourable than their self-assessment had the highest mortality risk.

### Strengths and limitations

Strengths of this study include its large sample size (> 370,000 participants) and a median follow-up of almost 12 years. Risk indices are often developed in high-risk populations (e.g., in older individuals), focus on a single health outcome and studies are often limited by small sample sizes. The health cross-classification examined in this study might be applicable for risk stratification for a wide range of health outcomes, which could be examined in future studies.

Our research inevitably has limitations. There might be some misclassification in the reporting of medical illnesses that were used to determine health status. However, participants were asked to report illnesses that had been diagnosed by a doctor and these diagnoses were confirmed during a nurse-led interview. Nevertheless, there is the possibility of recall bias in the reporting of long-term and past conditions. Our study confirms the criterion validity of the objective health status classification as it was highly predictive of mortality and is for that reason used in life insurance. Future studies could further examine the validity and reliability of this classification using other data sources such as patient records. Regarding self-rated health, there could be differences by native language in evaluating fair self-rated health as positive or negative. However, most participants were born in the UK and such differences, if present, would likely be minimal. Mortality data from death certificates might have some misclassification in causes of death, especially between similar diseases. We also observed fewer deaths during follow-up for several specific causes of death, especially in individuals with poor self-rated health and favourable health status or excellent self-rated health and unfavourable health status. However, all-cause mortality is a more robust endpoint than cause-specific mortality and we found similar results across most outcomes.

### Generalisability

Compared with non-responders, UK Biobank participants were older, more likely to be female and more likely of higher socioeconomic status. They were also less likely to engage in unhealthy lifestyle behaviours, reported fewer medical illnesses compared with data from a nationally representative survey and all-cause mortality was 46.2% lower for 70–74-year-olds^28^. Although these findings show that participants in the UK Biobank differ from the UK general population across multiple dimensions, the implication of this healthy participation bias, including its magnitude and direction of effect, is uncertain. A recent empirical investigation comparing the UK Biobank with data from 18 prospective cohort studies with conventional response rates showed that the direction of risk factor associations were similar, although with differences in magnitude^29^. Empirical research examining the potential impact of unrepresentativeness on associations between health status, self-rated health and mortality is warranted, as has been done for lifestyle risk factors^30^. It is worth noting that most participants (79.6%) in our sample reported good to excellent self-rated health, which is comparable to estimates from the Office for National Statistics suggesting that >72% of people in England and Wales rated their health as good or very good^31^. Our findings cannot be extrapolated beyond the age range of 37 to 73 and future research could examine the cross-classification of health status and self-rated health for risk stratification in younger and older populations.

### Implications

Risk stratification is a key public health priority that is central to clinical decision making and resource allocation. Self-rated health and health status can be obtained through verbal interview, self-report or from medical records. The cross-classification between health status and self-rated health represents a straightforward metric for initial risk stratification, with applications to population health, clinical decision making and resource allocation. Our findings also highlight that self-rated health captures additional health-related information beyond medical diagnoses and should therefore be more widely assessed across settings to improves health outcomes.

## Supplementary Information

Supplementary Information.

## Data Availability

The data used are available to all bona fide researchers for health-related research that is in the public interest, subject to an application process and approval criteria. Study materials are publicly available online at http://www.ukbiobank.ac.uk.

## References

[1] DeSalvo KB, Bloser N, Reynolds K, He J, Muntner P (2006). Mortality prediction with a single general self-rated health question: A meta-analysis. J. Gen. Intern. Med..

[2] Idler EL, Benyamini Y (1997). Self-rated health and mortality: A review of twenty-seven community studies. J. Health Soc. Behav..

[3] Idler EL (2003). Discussion: Gender differences in self-rated health, in mortality, and in the relationship between the two. Gerontologist.

[4] Singh-Manoux A (2007). The association between self-rated health and mortality in different socioeconomic groups in the GAZEL cohort study. Int. J. Epidemiol..

[5] Young H, Grundy E, O'Reilly D, Boyle P (2010). Self-rated health and mortality in the UK: Results from the first comparative analysis of the England and Wales, Scotland, and Northern Ireland Longitudinal Studies. Popul. Trends.

[6] O'Reilly D, Rosato M, Patterson C (2005). Self reported health and mortality: Ecological analysis based on electoral wards across the United Kingdom. BMJ.

[7] Ferraro, K. F. Self-ratings of health among the old and the old-old. *J. Health Soc. Behav.* 377–383 (1980). 7204931

[8] Wu S (2013). The relationship between self-rated health and objective health status: A population-based study. BMC Public Health.

[9] Goldberg P, Guéguen A, Schmaus A, Nakache J, Goldberg M (2001). Longitudinal study of associations between perceived health status and self reported diseases in the French Gazel cohort. J. Epidemiol. Community Health.

[10] Lima-Costa MF, Cesar CC, Chor D, Proietti FA (2012). Self-rated health compared with objectively measured health status as a tool for mortality risk screening in older adults: 10-year follow-up of the Bambuí Cohort Study of Aging. Am. J. Epidemiol..

[11] ONS. *How do people rate their general health? An analysis of general health by disability and deprivation* <https://webarchive.nationalarchives.gov.uk/20160109203542/http:/www.ons.gov.uk/ons/dcp171776_353238.pdf> (2014).

[12] ONS. *Leading causes of death, UK*, <https://www.ons.gov.uk/peoplepopulationandcommunity/healthandsocialcare/causesofdeath/articles/leadingcausesofdeathuk/2001to2018> (2020).

[13] Bycroft C (2018). The UK Biobank resource with deep phenotyping and genomic data. Nature.

[14] Maxwell, J. M. *et al.* Multifactorial disorders and polygenic risk scores: Predicting common diseases and the possibility of adverse selection in life and protection insurance. *Ann. Act. Sci.* 1–16 (2020).

[15] Mutz J, Roscoe CJ, Lewis CM (2021). Exploring health in the UK Biobank: Associations with sociodemographic characteristics, psychosocial factors, lifestyle and environmental exposures. BMC Med..

[16] Hartzband P, Groopman J (2012). There is more to life than death. N. Engl. J. Med..

[17] Guggenheim JA, Williams C (2016). Childhood febrile illness and the risk of myopia in UK Biobank participants. Eye.

[18] Ganna A, Ingelsson E (2015). 5 year mortality predictors in 498 103 UK Biobank participants: A prospective population-based study. Lancet.

[19] Kaplan EL, Meier P (1958). Nonparametric estimation from incomplete observations. J. Am. Stat. Assoc..

[20] Cox DR (1972). Regression models and life-tables. J. R. Stat. Soc. Ser. B (Methodol.).

[21] Benjamini Y, Hochberg Y (1995). Controlling the false discovery rate: A practical and powerful approach to multiple testing. J. Roy. Stat. Soc. Ser. B (Methodol.).

[22] Svedberg P, Bardage C, Sandin S, Pedersen NL (2006). A prospective study of health, life-style and psychosocial predictors of self-rated health. Eur. J. Epidemiol..

[23] Bzostek S, Goldman N, Pebley A (2007). Why do Hispanics in the USA report poor health?. Soc. Sci. Med..

[24] Damián J, Pastor-Barriuso R, Valderrama-Gama E, de Pedro-Cuesta J (2015). Discordance between physician-rated health and an objective health measure among institutionalized older people. BMC Geriatr..

[25] Viljanen A (2021). Subjective and objective health predicting mortality and institutionalization: An 18-year population-based follow-up study among community-dwelling Finnish older adults. BMC Geriatr..

[26] DeSalvo KB, Muntner P (2011). Discordance between physician and patient self-rated health and all-cause mortality. Ochsner J..

[27] Giltay EJ, Vollaard AM, Kromhout D (2012). Self-rated health and physician-rated health as independent predictors of mortality in elderly men. Age Ageing.

[28] Fry A (2017). Comparison of sociodemographic and health-related characteristics of UK Biobank participants with those of the general population. Am. J. Epidemiol..

[29] Batty GD, Gale CR, Kivimäki M, Deary IJ, Bell S (2020). Comparison of risk factor associations in UK Biobank against representative, general population based studies with conventional response rates: Prospective cohort study and individual participant meta-analysis. BMJ.

[30] Stamatakis E (2021). Is cohort representativeness passé? Poststratified associations of lifestyle risk factors with mortality in the UK biobank. Epidemiology.

[31] ONS*. General Health in England and Wales: 2011 and comparison with 2001, *<https://www.ons.gov.uk/peoplepopulationandcommunity/healthandsocialcare/healthandwellbeing/articles/generalhealthinenglandandwales/2013-01-30> (2013).

