# Supplementary material for: Cross-classification between self-rated health and health status: longitudinal analyses of all-cause mortality and leading causes of death in the UK
Source: Sci Rep. 2022 Jan 10;12:459. doi: 10.1038/s41598-021-04016-x (PMC8748682; doi:10.1038/s41598-021-04016-x)
Supplement: Supplementary file 1 — Supplementary Information. [file 41598_2021_4016_MOESM1_ESM.pdf]

Supplementary material

The following material accompanies the article:

Cross-classification between self-rated health and health status: longitudinal analyses of all-cause mortality and leading causes of death in the UK

Julian Mutz[1*] and Cathryn M. Lewis[1,2]

Affiliations:

1. Social, Genetic and Developmental Psychiatry Centre, Institute of Psychiatry, Psychology & Neuroscience, King's College London, London, United Kingdom
2. Department of Medical and Molecular Genetics, Faculty of Life Sciences & Medicine, King's College London, London, United Kingdom

Supplement 1: Hypotheses

Full health cross-classification

Full health cross-classification

Health status	Self-rated health: Poor	Fair	Good	Excellent
Favourable	?	?	↑	↑↑
Unfavourable	↓↓	↓	?	?

Simplified health cross-classification

Simplified health cross-classification

Health status	Self-rated health: Poor/Fair	Good/Excellent
Favourable	?	↑
Unfavourable	↓	?

Supplement 2: Additional results

Supplement figure 1. Study population

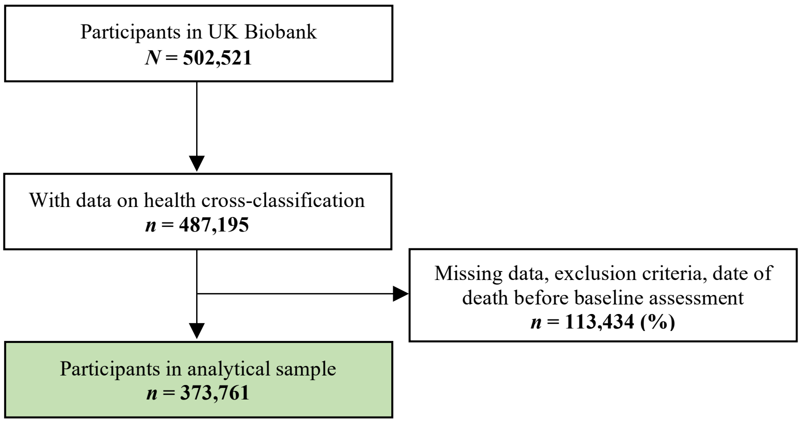

Supplement figure 1. Flowchart of study sample.

Supplement table 1. Sample characteristics

Supplement table 1. Sample characteristics

	Full sample (N=502521)	Analytical sample (N=373761)
**Age**		
Mean (SD)	56.53 (8.10)	56.02 (8.08)
**Sex**		
Female	273394 (54.4%)	193541 (51.8%)
Male	229127 (45.6%)	180220 (48.2%)
**Ethnicity**		
White	472711 (94.1%)	357509 (95.7%)
Mixed-race	2958 (0.6%)	2117 (0.6%)
Black	8061 (1.6%)	4877 (1.3%)
Asian	9882 (2.0%)	5551 (1.5%)
Chinese	1574 (0.3%)	1023 (0.3%)
Other	4558 (0.9%)	2684 (0.7%)
Prefer not to answer	1662 (0.3%)	
Do not know	217 (0.0%)	
Missing	898 (0.2%)	
**Highest qualification**[1]		
None	85274 (17.0%)	49621 (13.3%)
O levels/GCSEs/CSEs	132086 (26.3%)	99810 (26.7%)
A levels/NVQ/HND/HNC[1]	113859 (22.7%)	88033 (23.6%)
Degree	161168 (32.1%)	136297 (36.5%)
Prefer not to answer	5493 (1.1%)	
Missing	4641 (0.9%)	
**Household income**[2]		
Very low	97205 (19.3%)	78241 (20.9%)
Low	108177 (21.5%)	94386 (25.3%)
Medium	110774 (22.0%)	99882 (26.7%)
High	86269 (17.2%)	79742 (21.3%)
Very high	22930 (4.6%)	21510 (5.8%)
Prefer not to answer	49848 (9.9%)	
Do not know	21305 (4.2%)	
Missing	6013 (1.2%)	
**Walking** (days/week)[3]		
Mean (SD)	5.39 (1.93)	5.36 (1.95)
Prefer not to answer	979 (0.19%)	
Do not know	6687 (1.33%)	
Unable to walk	1929 (0.38%)	
Missing	874 (0.17%)	
**Moderate activity** (days/week)[3]		
Mean (SD)	3.63 (2.33)	3.57 (2.33)
Prefer not to answer	2273 (0.45%)	
Do not know	24120 (4.8%)	
Missing	878 (0.17%)	
**Vigorous activity** (days/week)[3]		
Mean (SD)	1.84 (1.96)	1.86 (1.94)
Prefer not to answer	4116 (0.82%)	
Do not know	22582 (4.49%)	
Missing	878 (0.17%)	
**Smoking status**		
Never	273528 (54.4%)	204457 (54.7%)
Former	173064 (34.4%)	131186 (35.1%)
Current	52979 (10.5%)	38118 (10.2%)
Prefer not to answer	2059 (0.4%)	
Missing	891 (0.2%)	
**Alcohol intake frequency**		
Never	40645 (8.1%)	25358 (6.8%)
Special occasions	58011 (11.5%)	38719 (10.4%)
1-3/month	55856 (11.1%)	41310 (11.1%)
1-2/week	129294 (25.7%)	96316 (25.8%)
3-4/week	115443 (23.0%)	91046 (24.4%)
Daily/almost daily	101770 (20.3%)	81012 (21.7%)
Prefer not to answer	605 (0.1%)	
Missing	897 (0.2%)	

Note: SD = standard deviation; GCSEs = general certificate of secondary education; CSE = certificate of secondary education; NVQ = national vocational qualification; HND = higher national diploma; HNC = higher national certificate. [1]also includes 'other professional qualifications'. [2]Annual household income groups: very low (<£18 000), low (£18 000–30 999), middle (£31 000–51 999), high (£52 000–100 000) and very high (>£100 000). [3]number of days per week engaging in these activities for 10+ minutes continuously.

Supplement table 2. Average number of illnesses

Supplement table 2. Number of illnesses by health cross-classification

Self-rated health	Health status	Mean	SD
Excellent	Favourable	0.73	0.99
Good	Favourable	1.19	1.27
Excellent	Unfavourable	2.26	1.43
Fair	Favourable	1.80	1.54
Good	Unfavourable	2.88	1.70
Poor	Favourable	2.52	1.81
Fair	Unfavourable	3.73	2.09
Poor	Unfavourable	4.93	2.70

Note: SD = standard deviation.

Supplement figure 2. Number of illnesses by health cross-classification

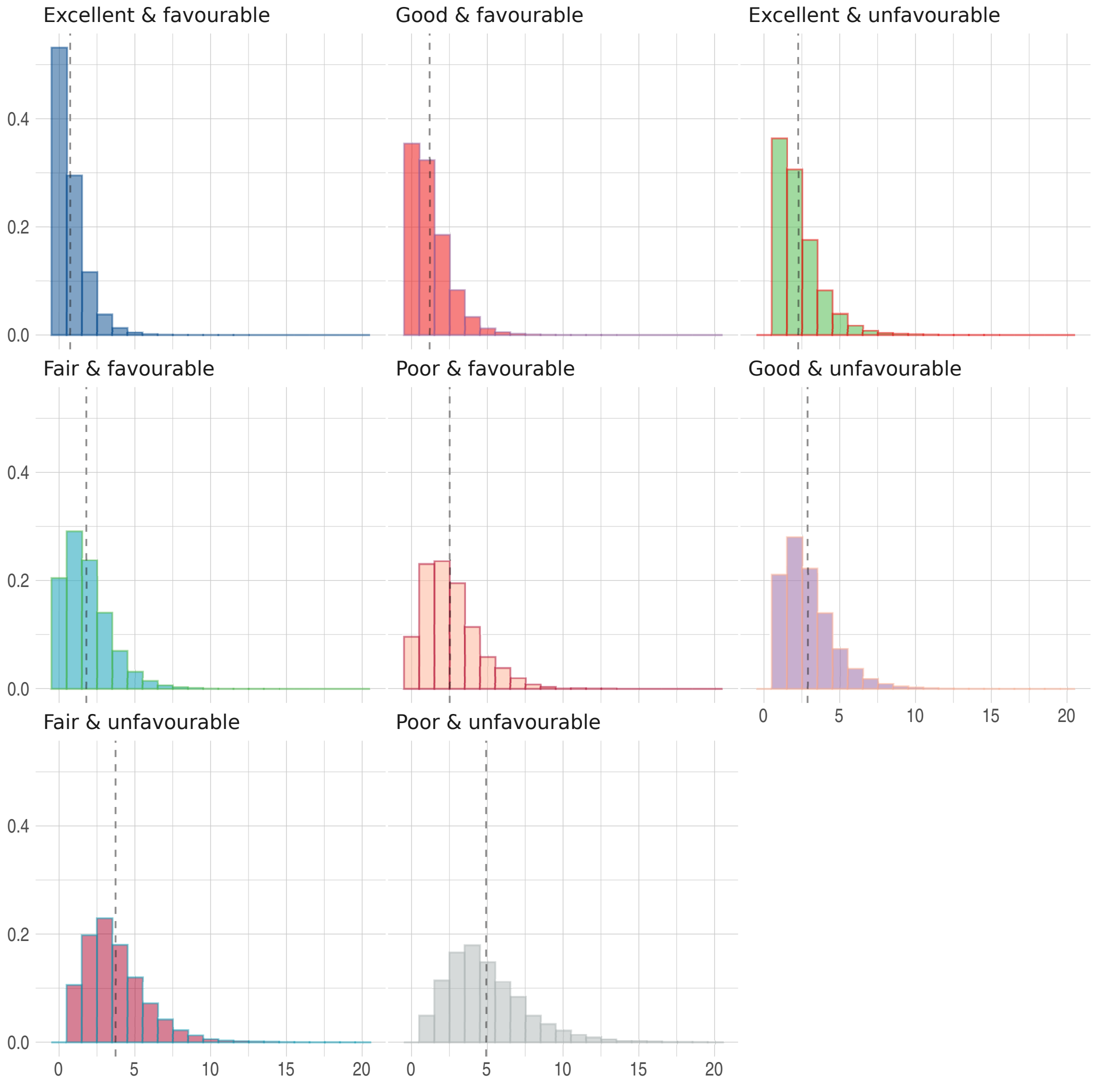

Supplement figure 2. Number of illnesses by health cross-classification. 9 observations were above graph maximum of 20 illnesses (5 poor & unfavourable, 3 fair & unfavourable and 1 excellent & unfavourable).

Supplement figure 3. Non-cancer illnesses

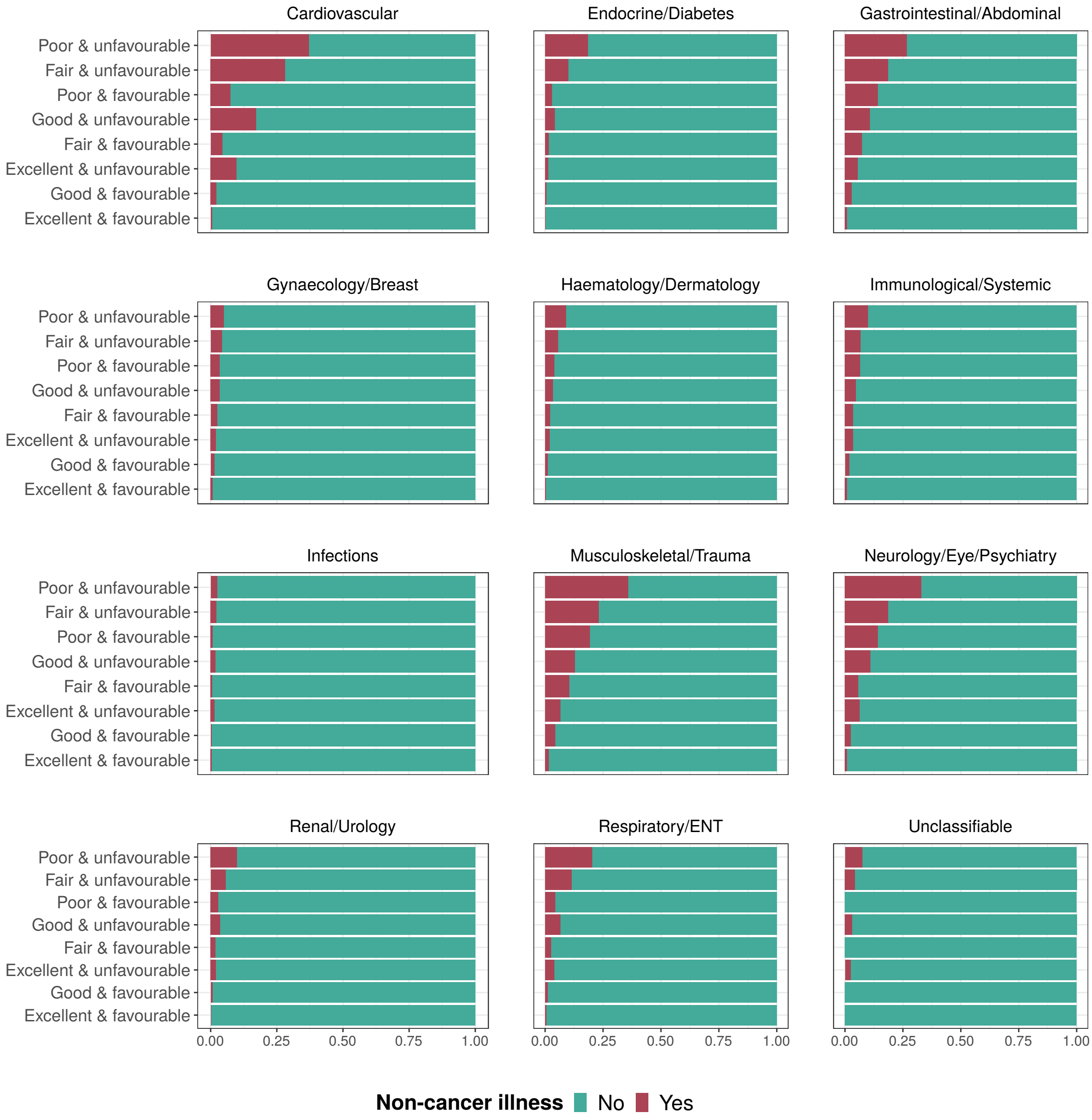

Supplement figure 3. Non-cancer illnesses by health cross-classification. Proportions represent individuals with at least one non-cancer illness within each broader illness group.

Supplement figure 4. Cancer illnesses

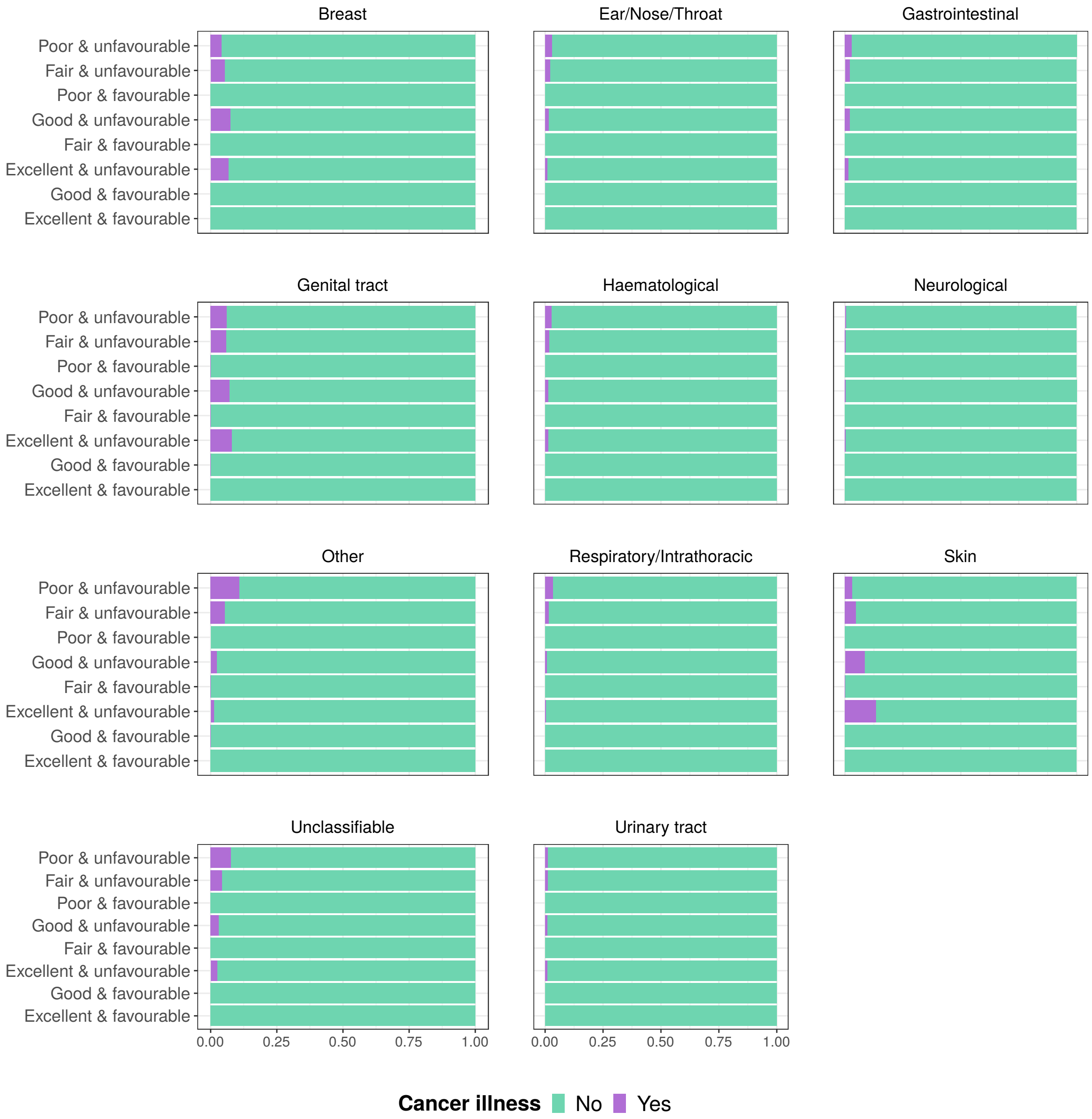

Supplement figure 4. Cancer illnesses by health cross-classification. Proportions represent individuals with at least one cancer illness within each broader illness group.

Supplement table 3. Hazard ratios health status and all-cause mortality

Supplement table 3. Cox proportional hazards model: health status and all-cause mortality

	Model 1			Model 2			Model 3		
Health status	HR	95% CI		HR	95% CI		HR	95% CI	
Healthy	Ref	-	-	Ref	-	-	Ref	-	-
Unhealthy	2.05	1.99	2.10	1.94	1.89	1.99	1.85	1.80	1.90

Note: HR = hazard ratio; CI = confidence interval. Model 1 – unadjusted; Model 2 – adjusted for sociodemographic characteristics; Model 3 – additionally adjusted for lifestyle factors. All Bonferroni-adjusted *P*-values < 0.001.

Supplement table 4. Hazard ratios self-rated health and all-cause mortality

Supplement table 4. Cox proportional hazards model: self-rated health and all-cause mortality

	Model 1			Model 2			Model 3		
Health status	HR	95% CI		HR	95% CI		HR	95% CI	
Excellent	Ref	-	-	Ref	-	-	Ref	-	-
Good	1.32	1.27	1.39	1.30	1.25	1.36	1.25	1.20	1.31
Fair	2.47	2.36	2.59	2.31	2.20	2.42	2.07	1.97	2.18
Poor	5.79	5.47	6.12	5.17	4.88	5.48	4.25	4.00	4.51

Note: HR = hazard ratio; CI = confidence interval. Model 1 – unadjusted; Model 2 – adjusted for sociodemographic characteristics; Model 3 – additionally adjusted for lifestyle factors. All Bonferroni-adjusted *P*-values < 0.001.

Supplement figure 5. Model 3 Cox PH survival probabilities

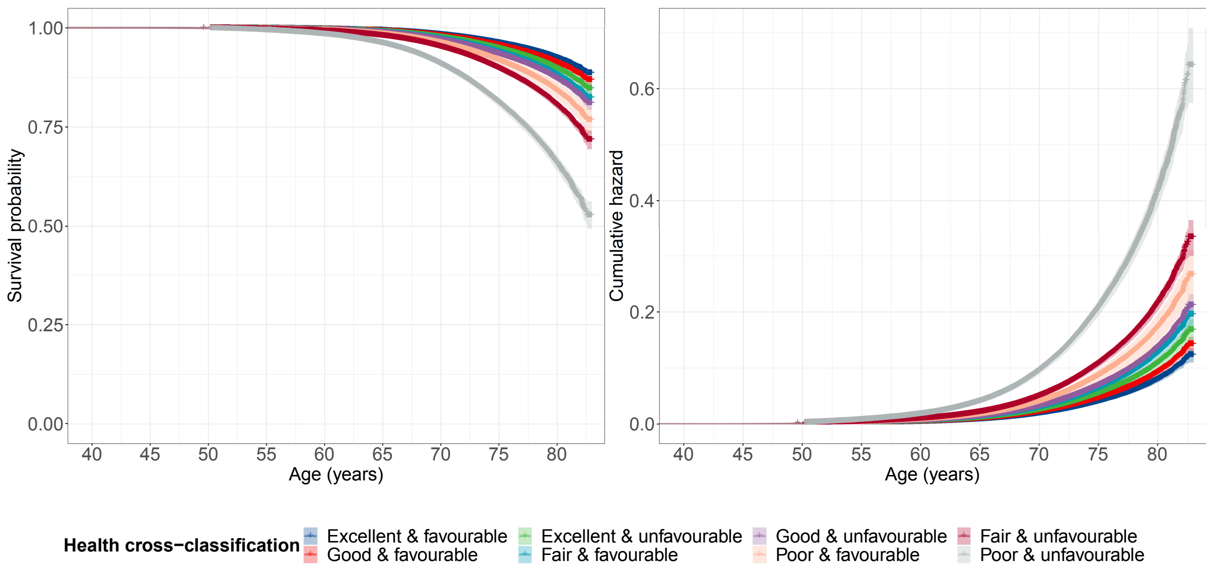

Supplement figure 5. Cox proportional hazard survival probabilities and cumulative hazards for all-cause mortality. Estimates from Model 3 adjusted for sex, ethnicity, highest qualification, household income, smoking status, alcohol intake and physical activity. 89 observations were above graph maximum of age 83. Figures show continuous covariates fixed at their mean value and categorical covariates fixed at the largest group.

Supplement figure 6. Survival curves cause-specific mortality

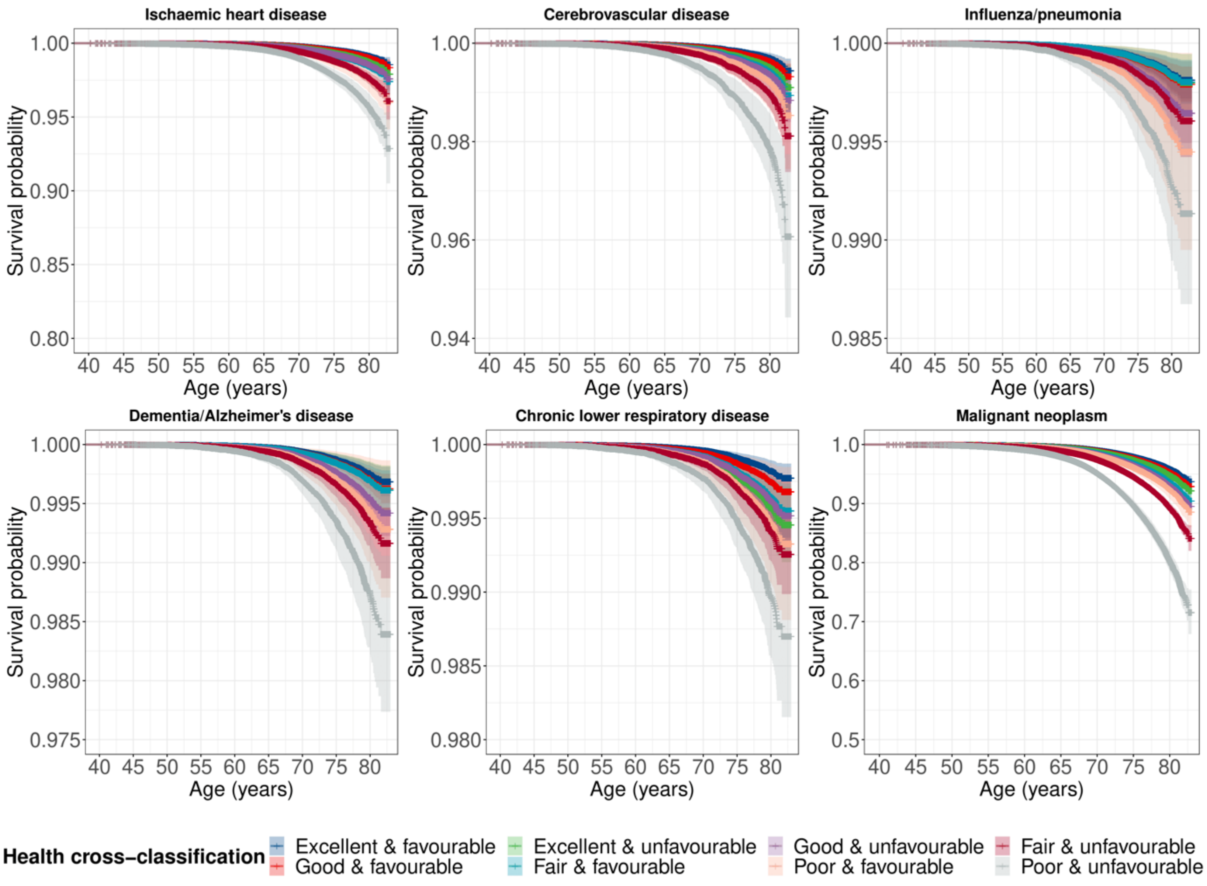

Supplement figure 6. Cox proportional hazard survival curves for cause-specific mortality.

Supplement table 5. Hazard ratios cause specific mortality

Results presented below correspond to Table 3 presented in the main body of the text but also include *p*-values. Results for which Bonferroni and Benjamini & Hochberg adjusted *p*-values differ are highlighted.

Supplement table 5. Cox proportional hazards model: cause-specific mortality

		Model 1					Model 2					Model 3				
Term		HR	95% CI		$p_{Bonf.}$	p_{BH}	HR	95% CI		$p_{Bonf.}$	p_{BH}	HR	95% CI		$p_{Bonf.}$	p_{BH}
**Ischaemic heart disease**																
Excellent	Favourable	Ref	-	-	-	-	Ref	-	-	-	-	Ref	-	-	-	-
Good	Favourable	1.21	1.02	1.43	0.557	0.029	1.19	1.01	1.41	0.889	0.044	1.14	0.96	1.35	>0.999	0.122
Excellent	Unfavourable	1.51	1.16	1.98	0.054	0.003	1.47	1.12	1.92	0.105	0.006	1.45	1.11	1.90	0.135	0.007
Fair	Favourable	2.16	1.77	2.63	<0.001	<0.001	2.03	1.66	2.48	<0.001	<0.001	1.81	1.48	2.21	<0.001	<0.001
Good	Unfavourable	1.84	1.55	2.18	<0.001	<0.001	1.77	1.49	2.10	<0.001	<0.001	1.68	1.42	2.00	<0.001	<0.001
Poor	Favourable	3.71	2.53	5.43	<0.001	<0.001	3.44	2.35	5.05	<0.001	<0.001	2.81	1.91	4.14	<0.001	<0.001
Fair	Unfavourable	3.38	2.85	4.01	<0.001	<0.001	3.10	2.60	3.68	<0.001	<0.001	2.76	2.32	3.29	<0.001	<0.001
Poor	Unfavourable	7.14	5.90	8.66	<0.001	<0.001	6.28	5.16	7.64	<0.001	<0.001	5.11	4.17	6.26	<0.001	<0.001
**Cerebrovascular diseases**																
Excellent	Favourable	Ref	-	-	-	-	Ref	-	-	-	-	Ref	-	-	-	-
Good	Favourable	1.25	0.94	1.66	>0.999	0.140	1.25	0.94	1.67	>0.999	0.140	1.22	0.91	1.62	>0.999	0.186
Excellent	Unfavourable	1.67	1.06	2.61	0.550	0.034	1.63	1.04	2.55	0.710	0.042	1.61	1.03	2.53	0.782	0.043
Fair	Favourable	2.13	1.52	3.00	<0.001	<0.001	2.09	1.48	2.95	0.001	<0.001	1.90	1.34	2.69	0.006	0.001
Good	Unfavourable	2.20	1.65	2.94	<0.001	<0.001	2.16	1.62	2.89	<0.001	<0.001	2.09	1.56	2.79	<0.001	<0.001
Poor	Favourable	3.24	1.61	6.54	0.022	0.002	3.16	1.56	6.39	0.029	0.002	2.64	1.30	5.36	0.150	0.010
Fair	Unfavourable	3.91	2.92	5.22	<0.001	<0.001	3.73	2.78	5.00	<0.001	<0.001	3.41	2.53	4.59	<0.001	<0.001
Poor	Unfavourable	9.49	6.93	13.01	<0.001	<0.001	8.74	6.34	12.07	<0.001	<0.001	7.19	5.15	10.03	<0.001	<0.001
**Influenza/pneumonia**																
Excellent	Favourable	Ref	-	-	-	-	Ref	-	-	-	-	Ref	-	-	-	-
Good	Favourable	1.22	0.82	1.82	>0.999	0.483	1.17	0.79	1.75	>0.999	0.604	1.11	0.75	1.66	>0.999	0.740
Excellent	Unfavourable	1.13	0.55	2.30	>0.999	0.857	1.09	0.53	2.22	>0.999	0.857	1.07	0.52	2.19	>0.999	0.857
Fair	Favourable	1.32	0.77	2.25	>0.999	0.483	1.19	0.70	2.04	>0.999	0.684	1.05	0.61	1.80	>0.999	0.857
Good	Unfavourable	2.17	1.46	3.24	0.003	<0.001	2.02	1.35	3.02	0.012	0.002	1.90	1.27	2.84	0.037	0.004
Poor	Favourable	4.13	1.72	9.91	0.031	0.003	3.63	1.51	8.74	0.085	0.008	2.96	1.22	7.18	0.339	0.028
Fair	Unfavourable	2.74	1.80	4.17	<0.001	<0.001	2.40	1.57	3.66	0.001	<0.001	2.12	1.38	3.25	0.013	0.002
Poor	Unfavourable	6.92	4.36	10.98	<0.001	<0.001	5.71	3.56	9.15	<0.001	<0.001	4.66	2.86	7.58	<0.001	<0.001
**Dementia/Alzheimer's disease**																
Excellent	Favourable	Ref	-	-	-	-	Ref	-	-	-	-	Ref	-	-	-	-
Good	Favourable	1.25	0.92	1.70	>0.999	0.219	1.24	0.91	1.68	>0.999	0.230	1.19	0.87	1.62	>0.999	0.337
Excellent	Unfavourable	1.28	0.76	2.17	>0.999	0.392	1.24	0.73	2.10	>0.999	0.440	1.23	0.72	2.07	>0.999	0.447
Fair	Favourable	1.44	0.96	2.15	>0.999	0.121	1.38	0.92	2.06	>0.999	0.179	1.23	0.82	1.85	>0.999	0.363
Good	Unfavourable	2.02	1.48	2.75	<0.001	<0.001	1.95	1.43	2.66	0.001	<0.001	1.85	1.35	2.53	0.003	<0.001
Poor	Favourable	2.87	1.30	6.32	0.186	0.019	2.79	1.26	6.16	0.235	0.021	2.29	1.03	5.09	0.861	0.072
Fair	Unfavourable	3.22	2.34	4.41	<0.001	<0.001	3.00	2.18	4.13	<0.001	<0.001	2.67	1.93	3.70	<0.001	<0.001
Poor	Unfavourable	7.00	4.91	9.99	<0.001	<0.001	6.35	4.42	9.14	<0.001	<0.001	5.16	3.54	7.51	<0.001	<0.001
**Chronic lower respiratory disease**																
Excellent	Favourable	Ref	-	-	-	-	Ref	-	-	-	-	Ref	-	-	-	-
Good	Favourable	1.50	1.07	2.09	0.382	0.020	1.46	1.04	2.04	0.584	0.029	1.42	1.01	1.98	0.904	0.043
Excellent	Unfavourable	2.52	1.58	4.03	0.002	<0.001	2.46	1.54	3.94	0.003	<0.001	2.41	1.51	3.86	0.005	<0.001
Fair	Favourable	2.38	1.60	3.54	<0.001	<0.001	2.19	1.47	3.26	0.002	<0.001	1.99	1.33	2.97	0.017	0.001
Good	Unfavourable	2.35	1.67	3.31	<0.001	<0.001	2.25	1.60	3.16	<0.001	<0.001	2.14	1.52	3.01	<0.001	<0.001
Poor	Favourable	4.01	1.88	8.55	0.007	<0.001	3.58	1.68	7.65	0.021	0.001	2.99	1.39	6.41	0.104	0.006
Fair	Unfavourable	4.09	2.90	5.76	<0.001	<0.001	3.68	2.60	5.21	<0.001	<0.001	3.30	2.33	4.69	<0.001	<0.001
Poor	Unfavourable	8.10	5.51	11.90	<0.001	<0.001	6.96	4.71	10.31	<0.001	<0.001	5.80	3.87	8.68	<0.001	<0.001
**Malignant neoplasm**																
Excellent	Favourable	Ref	-	-	-	-	Ref	-	-	-	-	Ref	-	-	-	-
Good	Favourable	1.19	1.10	1.29	<0.001	<0.001	1.18	1.09	1.27	<0.001	<0.001	1.14	1.06	1.23	0.016	0.001
Excellent	Unfavourable	1.31	1.15	1.49	0.001	<0.001	1.27	1.12	1.45	0.006	<0.001	1.26	1.11	1.44	0.009	<0.001
Fair	Favourable	1.81	1.65	1.99	<0.001	<0.001	1.72	1.57	1.89	<0.001	<0.001	1.55	1.41	1.71	<0.001	<0.001
Good	Unfavourable	1.84	1.70	1.99	<0.001	<0.001	1.78	1.65	1.93	<0.001	<0.001	1.71	1.58	1.85	<0.001	<0.001
Poor	Favourable	2.40	1.94	2.96	<0.001	<0.001	2.25	1.82	2.79	<0.001	<0.001	1.88	1.52	2.33	<0.001	<0.001
Fair	Unfavourable	3.20	2.96	3.46	<0.001	<0.001	2.96	2.74	3.21	<0.001	<0.001	2.68	2.48	2.91	<0.001	<0.001
Poor	Unfavourable	7.02	6.43	7.67	<0.001	<0.001	6.24	5.70	6.83	<0.001	<0.001	5.20	4.73	5.71	<0.001	<0.001

Note: HR = hazard ratio; CI = confidence interval; Bonf. = Bonferroni; BH = Benjamini & Hochberg. Model 1 – unadjusted; Model 2 – adjusted for sociodemographic characteristics; Model 3 – additionally adjusted for lifestyle factors.

Supplement table 6. Hazard ratios all-cause mortality stratified by sex

Supplement table 6. Cox proportional hazards model: all-cause mortality stratified by sex

		Model 1					Model 2					Model 3				
Term		HR	95% CI		$p_{Bonf.}$	p_{BH}	HR	95% CI		$p_{Bonf.}$	p_{BH}	HR	95% CI		$p_{Bonf.}$	p_{BH}
**Males**																
Excellent	Favourable	Ref	-	-	-	-	Ref	-	-	-	-	Ref	-	-	-	-
Good	Favourable	1.25	1.17	1.35	<0.001	<0.001	1.23	1.14	1.32	<0.001	<0.001	1.19	1.10	1.28	<0.001	<0.001
Excellent	Unfavourable	1.36	1.21	1.52	<0.001	<0.001	1.34	1.19	1.50	<0.001	<0.001	1.32	1.18	1.49	<0.001	<0.001
Fair	Favourable	1.92	1.76	2.09	<0.001	<0.001	1.82	1.66	1.98	<0.001	<0.001	1.65	1.51	1.80	<0.001	<0.001
Good	Unfavourable	1.79	1.66	1.92	<0.001	<0.001	1.73	1.61	1.86	<0.001	<0.001	1.67	1.55	1.80	<0.001	<0.001
Poor	Favourable	3.32	2.79	3.95	<0.001	<0.001	3.00	2.52	3.57	<0.001	<0.001	2.53	2.13	3.02	<0.001	<0.001
Fair	Unfavourable	3.08	2.86	3.31	<0.001	<0.001	2.88	2.67	3.10	<0.001	<0.001	2.64	2.45	2.85	<0.001	<0.001
Poor	Unfavourable	6.86	6.33	7.43	<0.001	<0.001	6.16	5.67	6.68	<0.001	<0.001	5.25	4.83	5.72	<0.001	<0.001
**Females**																
Excellent	Favourable	Ref	-	-	-	-	Ref	-	-	-	-	Ref	-	-	-	-
Good	Favourable	1.17	1.08	1.28	0.005	<0.001	1.18	1.08	1.28	0.004	<0.001	1.13	1.04	1.23	0.104	0.005
Excellent	Unfavourable	1.45	1.25	1.67	<0.001	<0.001	1.45	1.25	1.67	<0.001	<0.001	1.44	1.24	1.66	<0.001	<0.001
Fair	Favourable	1.70	1.52	1.89	<0.001	<0.001	1.69	1.52	1.89	<0.001	<0.001	1.50	1.34	1.67	<0.001	<0.001
Good	Unfavourable	1.93	1.77	2.11	<0.001	<0.001	1.93	1.77	2.11	<0.001	<0.001	1.83	1.68	2.00	<0.001	<0.001
Poor	Favourable	2.07	1.61	2.66	<0.001	<0.001	2.06	1.60	2.64	<0.001	<0.001	1.64	1.27	2.11	0.003	<0.001
Fair	Unfavourable	3.29	3.01	3.60	<0.001	<0.001	3.28	3.00	3.60	<0.001	<0.001	2.85	2.60	3.13	<0.001	<0.001
Poor	Unfavourable	6.64	5.97	7.39	<0.001	<0.001	6.59	5.91	7.34	<0.001	<0.001	5.11	4.57	5.72	<0.001	<0.001

Note: HR = hazard ratio; CI = confidence interval; Bonf. = Bonferroni; BH = Benjamini & Hochberg. Model 1 – unadjusted; Model 2 – adjusted for sociodemographic characteristics (excluding sex); Model 3 – additionally adjusted for lifestyle factors.

Supplement figure 7. Survival curve sensitivity analysis

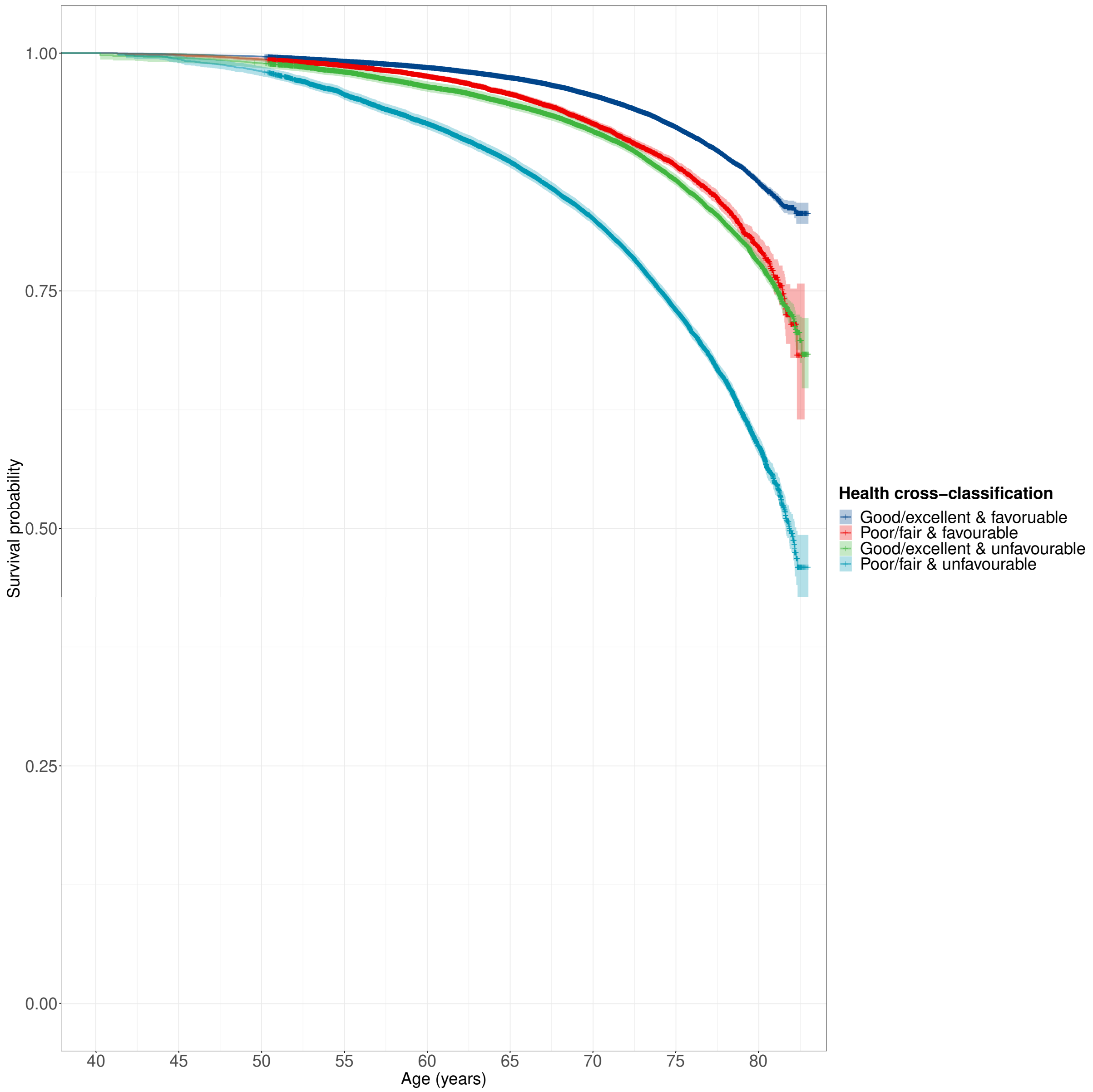

Supplement figure 7. Kaplan-Meier survival probabilities for all-cause mortality. Simplified health cross-classification. 89 observations were above graph maximum of age 83.

Supplement table 7. Hazard ratios all-cause mortality prior to 30 January 2020

Supplement table 7. Cox proportional hazards model: all-cause mortality prior to 30 January 2020

		Model 1			Model 2			Model 3		
Health cross-classification		HR	95% CI		HR	95% CI		HR	95% CI	
Excellent	Favourable	Ref	-	-	Ref	-	-	Ref	-	-
Good	Favourable	1.23	1.16	1.31	1.22	1.15	1.30	1.18	1.11	1.25
Excellent	Unfavourable	1.42	1.29	1.57	1.39	1.26	1.53	1.38	1.25	1.52
Fair	Favourable	1.88	1.75	2.03	1.79	1.67	1.93	1.61	1.50	1.73
Good	Unfavourable	1.87	1.76	1.99	1.81	1.70	1.92	1.73	1.63	1.84
Poor	Favourable	2.79	2.39	3.26	2.63	2.25	3.07	2.19	1.87	2.55
Fair	Unfavourable	3.32	3.12	3.52	3.08	2.89	3.27	2.78	2.61	2.95
Poor	Unfavourable	7.31	6.83	7.83	6.53	6.09	7.00	5.41	5.03	5.81

Note: HR = hazard ratio; CI = confidence interval. Model 1 – unadjusted; Model 2 – adjusted for sociodemographic characteristics; Model 3 – additionally adjusted for lifestyle factors. All Bonferroni-adjusted *P*-values < 0.001.

Supplement table 8. Hazard ratios cause specific mortality prior to 30 January 2020

Results for which statistical significance differed from the main analyses are highlighted (orange = no longer statistically significant, green = statistically significant only in the sensitivity analysis).

Supplement table 8. Cox proportional hazards model: cause-specific mortality prior to 30 January 2020

		Model 1					Model 2					Model 3				
Term		HR	95% CI		$p_{Bonf.}$	p_{BH}	HR	95% CI		$p_{Bonf.}$	p_{BH}	HR	95% CI		$p_{Bonf.}$	p_{BH}
**Ischaemic heart disease**																
Excellent	Favourable	Ref	-	-	-	-	Ref	-	-	-	-	Ref	-	-	-	-
Good	Favourable	1.17	0.98	1.39	>0.999	0.098	1.15	0.97	1.38	>0.999	0.121	1.11	0.92	1.32	>0.999	0.268
Excellent	Unfavourable	1.41	1.05	1.89	0.454	0.028	1.37	1.02	1.84	0.736	0.043	1.35	1.01	1.82	0.884	0.049
Fair	Favourable	2.09	1.69	2.58	<0.001	<0.001	1.98	1.60	2.45	<0.001	<0.001	1.75	1.41	2.17	<0.001	<0.001
Good	Unfavourable	1.75	1.46	2.10	<0.001	<0.001	1.69	1.41	2.03	<0.001	<0.001	1.61	1.34	1.93	<0.001	<0.001
Poor	Favourable	3.55	2.34	5.37	<0.001	<0.001	3.32	2.19	5.04	<0.001	<0.001	2.69	1.77	4.09	<0.001	<0.001
Fair	Unfavourable	3.37	2.81	4.04	<0.001	<0.001	3.12	2.59	3.74	<0.001	<0.001	2.76	2.29	3.33	<0.001	<0.001
Poor	Unfavourable	7.39	6.04	9.06	<0.001	<0.001	6.57	5.34	8.09	<0.001	<0.001	5.29	4.26	6.56	<0.001	<0.001
**Cerebrovascular diseases**																
Excellent	Favourable	Ref	-	-	-	-	Ref	-	-	-	-	Ref	-	-	-	-
Good	Favourable	1.28	0.93	1.75	>0.999	0.136	1.28	0.93	1.75	>0.999	0.136	1.24	0.90	1.70	>0.999	0.189
Excellent	Unfavourable	1.78	1.10	2.89	0.391	0.030	1.74	1.08	2.82	0.504	0.035	1.73	1.07	2.80	0.557	0.035
Fair	Favourable	2.23	1.54	3.24	<0.001	<0.001	2.17	1.50	3.16	0.001	<0.001	1.96	1.34	2.86	0.010	0.001
Good	Unfavourable	2.31	1.68	3.16	<0.001	<0.001	2.26	1.64	3.10	<0.001	<0.001	2.17	1.58	2.98	<0.001	<0.001
Poor	Favourable	2.63	1.13	6.15	0.531	0.035	2.56	1.09	6.00	0.631	0.037	2.11	0.90	4.96	>0.999	0.100
Fair	Unfavourable	4.12	3.00	5.66	<0.001	<0.001	3.91	2.84	5.39	<0.001	<0.001	3.53	2.55	4.89	<0.001	<0.001
Poor	Unfavourable	10.70	7.62	15.04	<0.001	<0.001	9.82	6.94	13.90	<0.001	<0.001	7.90	5.51	11.31	<0.001	<0.001
**Influenza/pneumonia**																
Excellent	Favourable	Ref	-	-	-	-	Ref	-	-	-	-	Ref	-	-	-	-
Good	Favourable	1.40	0.88	2.20	>0.999	0.227	1.35	0.85	2.13	>0.999	0.263	1.28	0.81	2.02	>0.999	0.346
Excellent	Unfavourable	1.51	0.72	3.18	>0.999	0.340	1.46	0.69	3.06	>0.999	0.357	1.44	0.68	3.02	>0.999	0.357
Fair	Favourable	1.64	0.91	2.94	>0.999	0.158	1.49	0.83	2.69	>0.999	0.255	1.31	0.72	2.37	>0.999	0.371
Good	Unfavourable	2.55	1.62	4.02	0.001	<0.001	2.38	1.50	3.76	0.004	0.001	2.24	1.42	3.55	0.012	0.001
Poor	Favourable	5.63	2.29	13.85	0.003	<0.001	4.98	2.02	12.30	0.011	0.001	4.06	1.63	10.09	0.054	0.005
Fair	Unfavourable	3.23	2.01	5.20	<0.001	<0.001	2.84	1.75	4.59	<0.001	<0.001	2.50	1.54	4.07	0.005	0.001
Poor	Unfavourable	8.63	5.16	14.45	<0.001	<0.001	7.17	4.24	12.13	<0.001	<0.001	5.86	3.41	10.07	<0.001	<0.001
**Dementia/Alzheimer's disease**																
Excellent	Favourable	Ref	-	-	-	-	Ref	-	-	-	-	Ref	-	-	-	-
Good	Favourable	1.26	0.91	1.75	>0.999	0.253	1.25	0.90	1.74	>0.999	0.265	1.20	0.86	1.67	>0.999	0.313
Excellent	Unfavourable	1.39	0.81	2.40	>0.999	0.291	1.35	0.78	2.34	>0.999	0.313	1.33	0.77	2.30	>0.999	0.318
Fair	Favourable	1.37	0.88	2.12	>0.999	0.253	1.31	0.84	2.04	>0.999	0.291	1.17	0.75	1.83	>0.999	0.483
Good	Unfavourable	1.95	1.39	2.73	0.002	<0.001	1.88	1.34	2.64	0.005	0.001	1.79	1.28	2.51	0.016	0.002
Poor	Favourable	3.37	1.52	7.48	0.058	0.006	3.26	1.47	7.26	0.079	0.007	2.69	1.21	6.03	0.332	0.028
Fair	Unfavourable	3.20	2.27	4.50	<0.001	<0.001	2.99	2.12	4.22	<0.001	<0.001	2.66	1.88	3.77	<0.001	<0.001
Poor	Unfavourable	6.79	4.61	9.99	<0.001	<0.001	6.18	4.17	9.17	<0.001	<0.001	5.04	3.35	7.58	<0.001	<0.001
**Chronic lower respiratory disease**																
Excellent	Favourable	Ref	-	-	-	-	Ref	-	-	-	-	Ref	-	-	-	-
Good	Favourable	1.40	0.99	1.99	>0.999	0.065	1.37	0.96	1.94	>0.999	0.084	1.34	0.95	1.91	>0.999	0.099
Excellent	Unfavourable	2.16	1.30	3.60	0.062	0.005	2.12	1.27	3.52	0.080	0.006	2.08	1.25	3.46	0.102	0.007
Fair	Favourable	2.27	1.50	3.44	0.002	<0.001	2.09	1.38	3.18	0.011	0.001	1.95	1.28	2.97	0.041	0.003
Good	Unfavourable	2.18	1.53	3.11	<0.001	<0.001	2.09	1.46	2.99	0.001	<0.001	2.02	1.41	2.89	0.003	<0.001
Poor	Favourable	3.27	1.39	7.74	0.144	0.009	2.93	1.24	6.94	0.309	0.018	2.55	1.07	6.07	0.722	0.040
Fair	Unfavourable	3.87	2.71	5.54	<0.001	<0.001	3.51	2.44	5.04	<0.001	<0.001	3.25	2.25	4.68	<0.001	<0.001
Poor	Unfavourable	7.45	4.96	11.20	<0.001	<0.001	6.49	4.28	9.82	<0.001	<0.001	5.65	3.68	8.66	<0.001	<0.001
**Malignant neoplasm**																
Excellent	Favourable	Ref	-	-	-	-	Ref	-	-	-	-	Ref	-	-	-	-
Good	Favourable	1.23	1.13	1.33	<0.001	<0.001	1.22	1.12	1.32	<0.001	<0.001	1.18	1.08	1.28	0.003	<0.001
Excellent	Unfavourable	1.31	1.14	1.51	0.003	<0.001	1.28	1.11	1.47	0.012	0.001	1.27	1.10	1.46	0.019	0.001
Fair	Favourable	1.85	1.67	2.05	<0.001	<0.001	1.77	1.60	1.96	<0.001	<0.001	1.59	1.44	1.76	<0.001	<0.001
Good	Unfavourable	1.86	1.70	2.02	<0.001	<0.001	1.80	1.65	1.96	<0.001	<0.001	1.73	1.58	1.88	<0.001	<0.001
Poor	Favourable	2.36	1.87	2.97	<0.001	<0.001	2.23	1.76	2.81	<0.001	<0.001	1.86	1.47	2.35	<0.001	<0.001
Fair	Unfavourable	3.28	3.01	3.57	<0.001	<0.001	3.05	2.80	3.33	<0.001	<0.001	2.76	2.53	3.01	<0.001	<0.001
Poor	Unfavourable	7.12	6.47	7.84	<0.001	<0.001	6.37	5.78	7.02	<0.001	<0.001	5.30	4.79	5.86	<0.001	<0.001

Note: HR = hazard ratio; CI = confidence interval; Bonf. = Bonferroni; BH = Benjamini & Hochberg. Model 1 – unadjusted; Model 2 – adjusted for sociodemographic characteristics; Model 3 – additionally adjusted for lifestyle factors.
